# Immunolocalization of steroidogenic enzymes in the vaginal mucous of *Galea spixii* during the estrous cycle

**DOI:** 10.1186/s12958-017-0248-3

**Published:** 2017-04-24

**Authors:** Amilton Cesar dos Santos, Alan James Conley, Moacir Franco de Oliveira, Gleidson Benevides Oliveira, Diego Carvalho Viana, Antônio Chaves de Assis Neto

**Affiliations:** 1 Department of Surgery, School of Veterinary Medicine and Animal Science, University of São Paulo, Av. Prof. Dr. Orlando Marques de Paiva, 87 ZC 05508-270 São Paulo, Brazil; 2 Population Health & Reproduction, School of Veterinary Medicine, University of California, Davis, 95616 USA; 3 Department of Veterinary Medicine, Universidade Federal Rural do Semiárido, Mossoró, 59625-900 Brazil

**Keywords:** Androgens, Cytochrome P450, Estrogens, Estrous cycle, Vaginal epithelium

## Abstract

**Background:**

The synthesis of sex steroids is controlled by several enzymes such as17α-hydroxylase cytochrome P450 (P450c17) catalyzing androgen synthesis and aromatase cytochrome P450 (P450arom) catalyzing estrogen synthesis, both of which must complex with the redox partner NADPH-cytochrome P450 oxidoreductase (CPR) for activity. Previous studies have identified expression of steroidogenic enzymes in vaginal tissue, suggesting local sex steroid synthesis. The current studies investigate P450c17, P450aromatase and CPR expression in vaginal mucosa of *Galea spixii* (Spix cavy) by immuno-histochemical and western immunoblot analyses.

**Methods:**

Stages of estrous cyclicity were monitored by vaginal exfoliative cytology. After euthanasia, vaginal tissues were retrieved, fixed and frozen at diestrus, proestrus, estrus and metestrus. The ovaries and testis were used as positive control tissues for immunohistochemistry.

**Results:**

Data from cytological study allowed identification of different estrous cycle phases. Immunohistochemical analysis showed different sites of expression of steroidogenic enzymes along with tissue response throughout different phases of the estrous cycle. However, further studies are needed to characterize the derived hormones synthesized by, and the enzymes activities associated with, vaginal tissues.

**Conclusion:**

Current results not only support the expression of enzymes involved in sex steroid synthesis in the wall of the vagina, they also indicate that expression changes with the stage of the cycle, both the levels and types of cells exhibiting expression. Thus, changes in proliferation of vaginal epithelial cells and the differentiation of the mucosa may be influenced by local steroid synthesis as well as circulating androgens and estrogens.

## Background

*Galea spixii* (Spix cavy) are rodents of the Caviidae family and subfamily Caviinae [1] that live in backland vegetation of the Brazilian northeastern region [2]. Most animals are bred in captivity for food [3, 4], in part to help preserve the species which is on the endangered list of the International Union of Conservation of Nature [5]. Spix cavy has also been employed as experimental models in research on placental function [2, 6]. Based on vaginal exfoliative cytology, the estrous cycle lasts 14–19 days and the vaginal epithelium undergoes variations related to each estrous cycle phases [4] in concert with ovarian follicular development and ovulation [7].

Laboratory rodents including mice, rats and guinea pigs are commonly used as experimental models since they have a relatively short estrous cycle and gestation length, and are easy to handle [8–11]. Guinea pigs (*Cavia porcellus*), like Spix cavy were employed in some of the earliest studies elucidating morphological and physiological changes in the reproductive tract and vagina during reproductive cyclicity [12–15]. Moreover, the biology of the vaginal mucosa has clinical significance. For instance, atrophy of the vaginal epithelium in post-menopausal women can have serious consequences for quality of life [16] requiring therapies aimed at restoring estrogenic stimulation lost as a result of decreased ovarian synthesis [17]. Though many treatment strategies employ local application of estrogens themselves [18], others have found success using dehydroepiandrosterone (DHEA) [19]. DHEA has little bioactivity of its own, but seems to provide benefits similar to estrogens [19], suggesting it may be used as a substrate for estrogen synthesis in the vaginal wall itself. Indeed, data has been reported that supports this possibility, specifically demonstrating the expression of steroidogenic enzymes in the vaginal epithelium [16, 20–22].

Therefore, the current investigation was undertaken to begin to define changes in the vaginal mucosa of Spix cavy and the potential expression of key steroidogenic enzymes such as 17α-hydroxylase/17,20-lyase (P450c17) [23, 24]; and aromatase (P450arom) [25, 26]; cytochromes P450 that are required for androgen and estrogen synthesis respectively.

## Methods

### Animals

Twelve adult (>6 months of age) females (*n* = 3 at each phase of the estrous cycle) were housed in outside enclosures (2.5 × 2.5 m wide; 2.2 m high) and fed a variety of fruits, grass, maize grains and a commercial rabbit diet with access to water *ad libitum*.

All were reared in captivity at the Wild Animals Multiplication Center of the Universidade Federal Rural do Semiárido, campus Mossoró RN Brazil (IBAMA 2028236/2008). Animal care and use conformed to protocols approved by the Committee of Bioethics of the Faculty of Veterinary Medicine and Animal Science of the Universidade de São Paulo (Protocol 2400/2011).

### Exfoliative vaginal cytology

Reproductive cycles were monitored using exfoliative vaginal cytology. Vaginal smears were collected daily with sterile cotton swabs and the biological material was deposited on glass slides stained with panoptic fast test, following instructions by the manufacturer (Laborclin®, Vargem Grande/Pinhais PR Brazil). Two full cycles were monitored for each animal prior to euthanasia and tissue collection, analyzed and photodocumented by light microscopy. The predominant cells in each smear were classified as superficial, large and small intermediate, parabasal and neutrophils throughout the cycle. Then the estrous cycles phases were characterized according the different cell types present in each phase [4, 7, 12, 13].

### Collection of material for immunohistochemistry

At each stage of the estrous cycle (estrus, metestrus, diestrus and proestrus), females were anesthetized with xylazine hydrochloride (4 mg/kg/IM) plus ketamine hydrochloride (60 mg/kg/IM) and then euthanized with sodium thiopental (2.5% 60 mg/kg). After euthanasia, the genital organs were then exposed and samples of tissues from the vaginal tube were retrieved in each phase of the cycle along with ovaries used as positive control tissue. Vaginal tissues were fixed in 4% buffered paraformaldehyde. Samples were dehydrated (ethanol series, from 50 to 100%), diaphanized in xylol and paraffin embedded. Additional tissue samples for western blotting were frozen at -80 °C.

### Immunohistochemistry

Tissue sections (5 μm, Leica RM2165) were placed in buffer at 70 °C for 3 h, diaphanized in xylol, re-hydrated and rinsed with distilled water, then heated at 90 °C immersed in a buffer citrate solution in a micro-oven. Endogenous peroxidase activity was blocked by hydrogen peroxide (H_2_O_2_, 3%) and blocked with normal horse serum (Vector Labs, Burlingame, CA, USA). Sections were incubated with primary antisera raised against P450arom anti-rabbit/mouse (1/400- ab18995, Abcam, Cambridge, MA, USA), NADPH-cytochrome P450 oxido-reductase (CPR) anti-mouse, rat, sheep, rabbit, guinea pig, hamster, cow, dog, human, pig, monkey (1/200- ab13513, Abcam, Cambridge, MA, USA), and P450c17 (1/200- Dr. Alan J. Conley, UC, Davis, California, USA), for 16 h. The primary antiserum was omitted for negative controls. Sections were rinsed, incubated with secondary anti-mouse/rabbit antisera ready-to-use (Immpress Universal Kit™,Vector Labs, Burlingame CA USA) followed by amplifier solution and developed with DAB (ImmPACT™ DAB, Vector Labs, Burlingame, CA USA). Sections were counter-stained with hematoxylin and mounted with coverslips. Images were captured with camera Olympus UTVO.5XC mounted on an Olympus BX61VS light microscope.

### Western immuno-blotting

The specificity of staining for P450c17 was verified by western immuno-blotting. Tissues were homogenized using a Polytron PT 3000 KINEMATICA^TM^ (Brinkman, Westbury, USA) in a hypotonic lysis buffer containing 50 mM potassium phosphate (pH 7.0), 0.3 M sucrose, 0.5 mM dithiothreitol (DTT), 1 mM ethylenediaminetetraacetic acid (EDTA, pH 8.0), 0.3 mM phenylmethylsulfonyl fluoride (PMSF), 10 mM NaF and phosphatase inhibitor cocktail (1: 100; Sigma-Aldrich). Protein concentration in homogenates was determined using Bradford’s method [27] according to the manufacturer (Protein Assay Kit; BIORAD, CA USA) and using albumin as a standard. Proteins (50ug) were denatured with Laemmli buffer (15% glycerin, 0.05 M Tris, 0.055 M bromophenol blue, 9% SDS) with 6% beta-mercaptoethanol (1:1), heated at 95 °C for 5 min. Proteins were resolved on one-dimensional SDS–PAGE minigels. The separated proteins were electro-transferred to immunoblot polyvinylidene difluoride membranes (PVDF-Bio-Rad Laboratories), at a constant current of 120 mA for 2 h, at 4 °C, in Tris-HCl buffer (Tris-HCl 12.5 mM, glycine, 1% SDS and 20% methanol). After transfer, the membranes were blocked with 5% non-fat dried milk in PBS-Tween 1% (PBS-T) for 2 h and incubated with a rabbit primary anti-bovine P450c17 (1:1000) anti-serum (Dr. Alan J. Conley, UC, Davis, CA USA) overnight at 4 °C.

On the following day the membranes were incubated with the secondary antibody anti-rabbit IgG-peroxidase conjugate (1/2000, BIORAD, CA USA) for 1 h at room temperature. The proteins were visualized by chemiluminescence (CHEMI-DOC®, BIORAD, CA USA) and images captured using commercial software (Image Lab® 4.01, BIORAD, CA USA). Molecular size of visualized bands was estimated from a commercial protein size ladder (Kaleidoscope®, BIORAD, CA USA). Loading was verified by incubation with a monoclonal mouse anti-β-actin antibody (Santa Cruz Biotechnology, Santa Cruz, CA, USA) diluted 1:10.000 in blocking solution for 2 h at room temperature. Membranes were then incubated with the secondary antibody anti-mouse IgG-peroxidase conjugate (1:2000, BIORAD, CA USA) for 1 h at room temperature. The proteins were visualized by chemiluminescence.

## Results

### Detecting the phases of the estrous cycle

Vaginal cytology allowed characterization of cycle stage. Superficial nucleated cells were predominant when sperm were also evident in the smear in those female co-housed with males and were therefore a reliable indication of estrus. Parabasal cells predominated during metestrus, small intermediate cells during diestrus which transitioned into large, superficial intermediate cells during proestrus. These changes were consistent from cycle to cycle and made it possible to predict the onset of estrus.

### Immunohistochemistry

The vaginal epithelium was typically stratified squamous with cell types that correlated with exfoliative cytological findings, varying among stages: a simple squamous a few cell layers in thickness during metestrus, a slight acceleration of proliferation and increased thickness during the diestrus, then, reaching several cell layers in thickness with cornification of the superficial layers and some loss of nuclei during proestrus. At estrus, the epithelium showed only one or a few layers of cornified cells and a low proliferation of intermediate and parabasal cells.

**Fig. 1:**
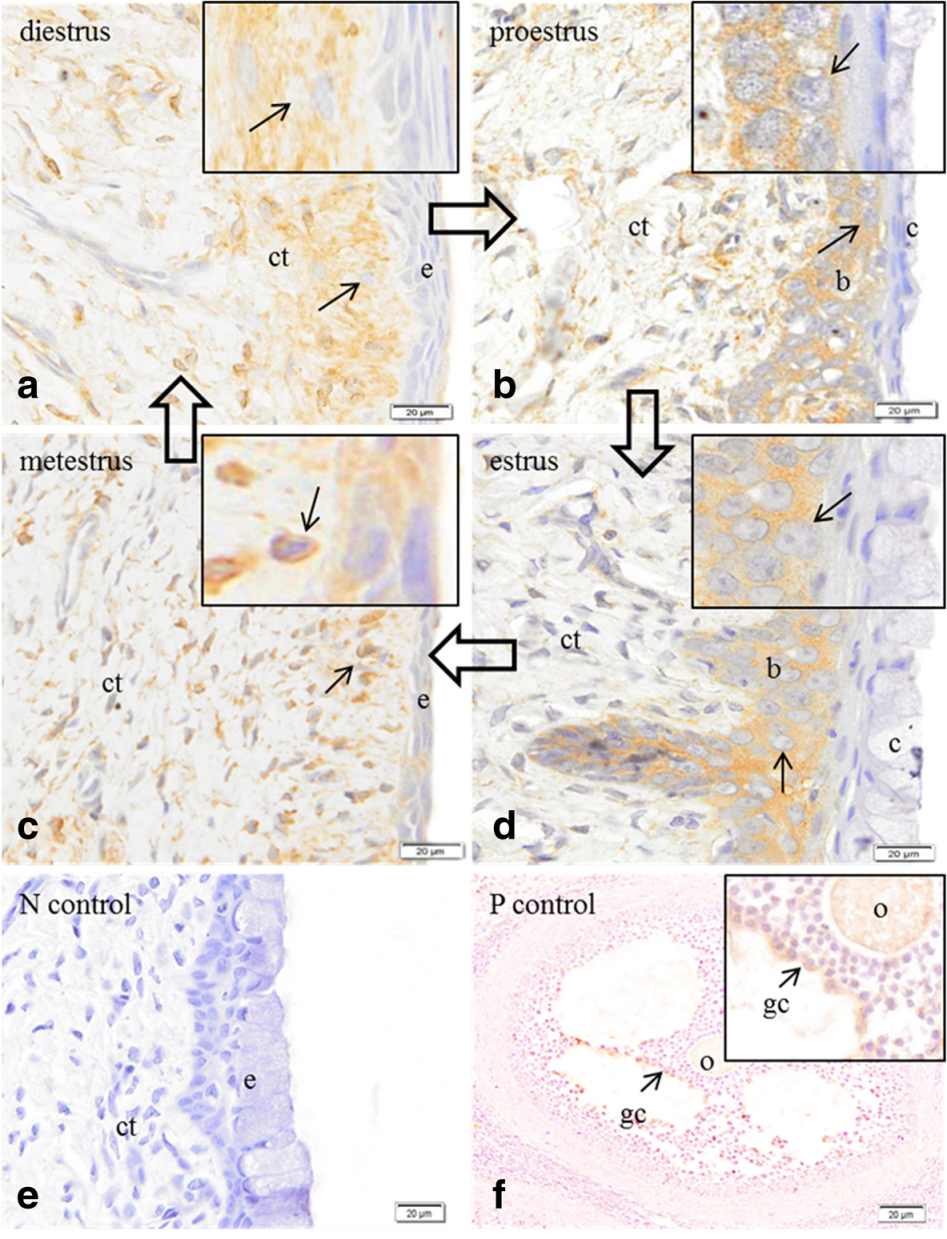
Immunolocalization of the enzyme cytochrome P450 aromatase in the vaginal epithelium and ovary of Spix cavy. **a** immunoreactivity in cells (*arrow*) of the connective tissue (*ct*); (*e*) epithelium in the vaginal wall at diestrus. **b** immunoreactivity in basal cells (*b*) of the epithelium in the vaginal wall (*c*) cornified cells of the epithelium; (*ct*) connective tissue in the vaginal wall at proestrus. **c** immunoreactivity in cells (*arrow*) of the connective tissue (*ct*); (*e*) epithelium in the vaginal wall at metestrus. **d** immunoreactivity in basal cells (*b*) of the epithelium; (*c*) cornified cells; (*ct*) connective tissue in the vaginal wall at estrus. **e** no immunoreactivity in the epithelium (*e*) or connective tissue (*ct*) in the absence of primary antiserum, negative control. **f** immunoreactivity in the cells of the granulosa layer (*gc*) of the ovary tissue; oocyte (*o*); positive control. Bars = 20 μm

Expression of P450arom was immunolocalized in cells of the connective tissue of the lamina propria during the diestrous phase (Fig. 1A); in the basal cells of the epithelium during proestrus (Fig. 1B); in basal cells of the epithelium in estrus (Fig. 1D); and in fewer scattered cells of the connective tissue during metestrus (Fig. 1C). No immunoreactivity was observed in the negative controls for the vaginal epithelium (Fig. 1E), and positive immunoreactivity was confined to the cells of the granulosa layer of antral follicles in ovarian tissue sections, further demonstrating specificity in the positive tissue controls (Fig. 1F).

**Fig. 2:**
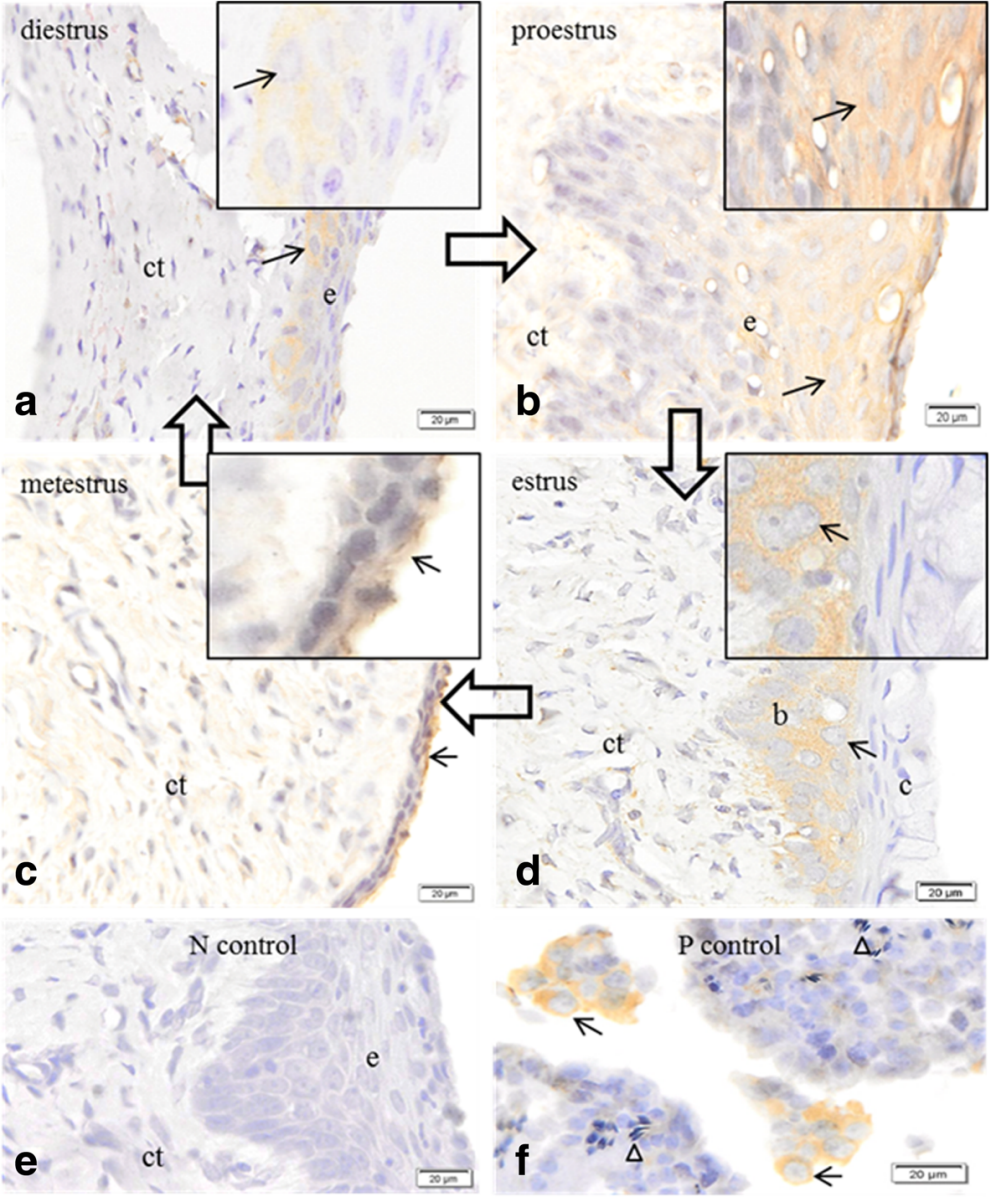
Immunolocalization of the enzyme cytochrome P450c 17 in the vaginal epithelium, and testes of Spix cavy. **a** immunoreactivity in basal cells (*arrow*) of the epithelium (*e*); (*ct*) connective tissue at diestrus. **b** immunoreactivity in surface cells (*arrow*) of the epithelium (*e*); (*ct*) connective tissue at proestrus. **c** immunoreactivity in epithelium cells (*arrow*); (*ct*) connective tissue at metestrus. **d** immunoreactivity (*arrow*) in basal cells (*b*); (*c*) cornified cells; (*ct*) connective tissue at estrus. **e** no immunoreactivity in epithelium cells (*e*) or connective tissue (*ct*). Negative control. **f** immunoreactivity in Leydig cells of the testicle (*arrow*). Spermatozoa (*arrowhead*). Positive control. Bars = 20 μm

Expression of P450c17 was detected in a few basal cells in the vaginal epithelium during diestrus (Fig. 2A); more superficial, surface cells during proestrus (Fig. 2B); and in basal cells of the epithelium in estrus (Fig. 2D). Immunoreactivity in the vaginal epithelial cells was weak during metestrus (Fig. 2C). No immunoreactivity was observed in the absence of the primary antisera (Fig. 2E). Immunoreactivity for P450c17 was restricted only to Leydig cells of testes (Fig. 2F).

**Fig. 3:**
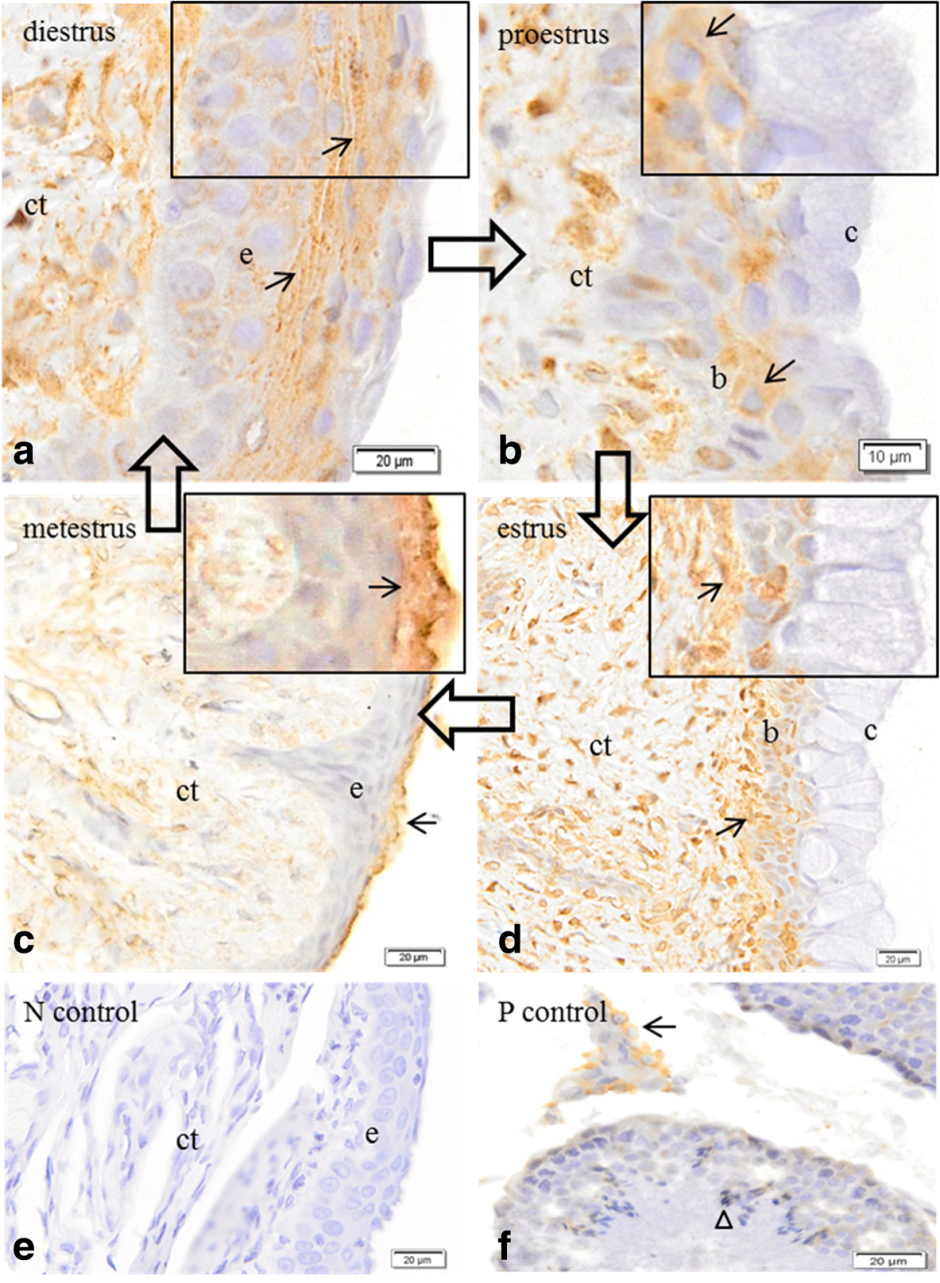
Immunolocalization of the enzyme NADPH cytochrome P450 reductase in the vaginal epithelium and testes of Spix cavy. **a** immunoreactivity in cells (*arrow*) of the epithelium (*e*) and connective tissue (*ct*) at diestrus. Bar = 20 μm. **b** immunoreactivity in the basal cells of the epithelium (*b*) and of the connective tissue (*ct*); (*c*) cornified surface cells of the epithelium at proestrus. Bar = 10 μm **c** immunoreactivity in cells (*arrows*) of the epithelium and connective tissue (*ct*); (*c*) cornified cells of the epithelium at metaestrus. **d** immunoreactivity in cells (*arrow*) of the connective tissue (*ct*) and basal cells (*b*) of the epithelium; cornified cells of the epithelium at estrus **e** no immunoreactivity in cells of the epithelium (*e*) or connective tissue (*ct*). Negative control. **f** immunoreactivity in Leydig cells of the testicle (*arrow*). Spermatozoa (*arrowhead*). Positive control. Bars = 20 μm

Immunolocalization of CPR indicated expression of this co-enzyme was present in the epithelium and in the cells of the connective tissue during diestrus (Fig. 3A); in the basal cells of the epithelium and in cells of the connective tissue in proestrus (Fig. 3B) and estrus (Fig. 3D); in the cells of the connective tissue and the epithelium during metestrus (Fig. 3C). Immunoreactivity was not detected in the absence of the primary antisera (Fig. 3E). Immunoreactivity was observed primarily in the Leydig cells of testes used as positive control (Fig. 3F).

### Western immuno-blotting

**Fig. 4:**
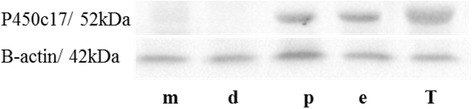
Western immuno-blotting test for enzyme cytochrome P450c17 in the vaginal tissue of Spix cavy in several phases of the estrous cycle. Metaestrus (*m*), diestrus (*d*), proestrus (*p*), estrus (*e*) and adult testicles (*T*)

Western immuno-blotting confirmed the expression of P450c17 in the vaginal epithelium during proestrus and estrus, but was not detectable during metestrus or diestrus (Fig. 4). Testicles of adult males used for positive control also were detected. Molecular size for P450c17 was slightly over 52 kDa and for endogenous control β actin was 42 kDa.

## Discussion

Current results not only support the expression of enzymes involved in sex steroid synthesis in the wall of the vagina, they also indicate that expression changes with the stage of the cycle, both the levels and types of cells exhibiting expression. Thus, changes in proliferation of vaginal epithelial cells and the differentiation of the mucosa may be influenced by local steroid synthesis as well as circulating estrogens. Lephart et al. [20] reported that aromatase activity in vaginal mucosa of rats was increased by testosterone. Testosterone is known to increase in serum and ovarian tissues in mice during proestrus [28], and vaginal mucosa expresses androgen receptors [16, 21] at levels that vary with age and menopausal status of women. Berman et al. [16] reported that the enzyme 5α-reductase which can convert testosterone into dihydrotestosterone is also expressed in the human vagina. It is possible that cycle stage-specific, vaginal expression of steroidogenic enzymes is an androgen-stimulated response. Furthermore, Iguchi et al. [29] demonstrated that treatment with testosterone, even administered concurrent with 5α-reductase and aromatase inhibitors, stimulated epithelial cell proliferation resulting in a marked increased thickness of the vaginal epithelium in ovariectomized mice. Therefore, it is likely that vaginal epithelial proliferation is stimulated directly by androgens.

Given the stimulatory response of vaginal epithelium to androgens, the possibility that androgen synthesis might occur locally within the vaginal epithelium itself assumes additional relevance. To the authors’ knowledge, no studies have been reported that examined the expression of the enzyme P450c17 in the vaginal tissue of any species. Confirmation that CPR, the redox partner protein for both P450c17 and P450arom, is also expressed in this tissue provides further support for local sex steroid synthesis. Although P450c17 expression in the vaginal wall of Spix cavy was less evident than aromatase and cytochrome P450 NADPH reductase, immuno-staining in positive controls tissues (Leydig cells), together with the apparent molecular size as expected by western immunoblot, supports its presence. Since levels were detectable only during the proestrous and estrous phases, local synthesis might coincide with vaginal proliferation and augment the effects of ovarian steroids. Equally, increased expression during proestrus might reflect regulation of P450c17 in vaginal epithelium by ovarian androgens. Further studies must be undertaken to quantify the levels of enzyme induced and the steroids synthesized.

With regard to the local production of androgens in the vaginal tissue, Berman et al. [16] reported for the first time that the enzyme 5α-reductase, which can convert testosterone into dihydrotestosterone, is expressed in association with androgen receptors in the human vagina. The authors also observed that the levels of androgen receptors varied according to age and menopausal status of women.

Proliferation of the vaginal epithelium is clearly stimulated by estrogens, and probably androgens, but their effects are modified by progestins [30] in a complex dose-dependent fashion, highly stimulatory at low levels but inhibitory at higher doses [31]. ***In Spix cavy, local synthesis of estrogens and/or androgen, as implied by steroidogenic enzyme expression, may induce or contribute to cyclic epithelial differentiation in vaginal tissue. Further studies, perhaps using local application of inhibitors of sex steroid synthesis may reveal the physiological importance of local steroid production in vaginal epithelium of Spix cavy, building on results of past studies in guinea pigs*** [30] ***and rats*** [32].

***Effects of vaginal dehydroepiandrostenedione application, vaginal testosterone, and tissue selective estrogen complexes have been promoted as promising new therapies in post-menopausal women; however, further studies are necessary to confirm their efficacy and safety*** [19]. ***Based on present results, the local synthesis of dehydroepiandrostenedione by P450c17 may be considered in further studies in other species including women.*** Notwithstanding potential species differences, Spix cavy may prove to be an excellent experimental model of relevance in studies on vaginal biology and health of post-menstrual women, women treated with aromatase inhibitors [17, 18] or other diseases of the female reproduction system [16, 25, 32–34]. These studies might include local concentrations of various steroids in multiple reproductive tissues, or perhaps the effects of local application of P450 enzyme inhibitors.

## Conclusions

Immunohistochemical analysis showed different sites of expression of sex steroidogenic enzymes along with tissue response throughout different phases of the estrous cycle. Results also indicate that expression changes with the stage of the cycle, both the levels and types of cells exhibiting expression. Thus, changes in proliferation of vaginal epithelial cells and the differentiation of the mucosa may be influenced by local steroid synthesis as well as circulating androgens and estrogens.

## Abbreviations

Arom: Aromatase
CPR: NADPH-cytochrome P450 oxidoreductase
DHEA: Dehydroepiandrosterone
